# Metacognitive Belief Profiles Across OCD Symptom Dimensions: A Systematic Review and Clinical Implications for Personalised Treatment

**DOI:** 10.3390/jcm15103586

**Published:** 2026-05-07

**Authors:** Vassilis Martiadis, Fabiola Raffone, Concetta Iaccarino, Emilia Carbone, Carmine De Simone, Clemente Purcaro, Miriam Olivola, Tommaso Barlattani, Domenico De Berardis, Francesca Pacitti

**Affiliations:** 1 Department of Mental Health, Azienda Sanitaria Locale Napoli 1 Centro, 80125 Naples, Italy; 2 Department of Psychiatry, University of Campania “Luigi Vanvitelli”, 80141 Naples, Italy; 3 Department of Biomedical and Clinical Sciences Luigi Sacco, University of Milan, 20157 Milan, Italy; 4 Department of Biotechnological and Applied Clinical Sciences (DISCAB), University of L’Aquila, 67100 L’Aquila, Italy; 5 Department of Mental Health, Asl Teramo, 64100 Teramo, Italy

**Keywords:** obsessive–compulsive disorder, metacognition, symptom dimensions, MCQ-30, thought-action fusion, beliefs about rituals, stop signals, metacognitive therapy, exposure and response prevention, personalised treatment

## Abstract

**Background/Objectives**: Although obsessive–compulsive disorder (OCD) is clinically heterogeneous, it is unclear whether specific metacognitive belief domains are differentially associated with particular symptom dimensions in adults with confirmed OCD. This systematic review synthesised the available clinical evidence and explored its implications for dimension-informed case formulation and treatment planning. **Methods**: In March 2026, PubMed/MEDLINE, Scopus, Cochrane CENTRAL and Google Scholar were searched without date or language restrictions. Eligible studies enrolled adults with a confirmed diagnosis of OCD, administered at least one validated metacognitive instrument (MCQ-30/65, TFI, TAF, BARI, SSQ or OBQ-based subscales) and reported associations with validated dimensional OCD measures. The review was preregistered on PROSPERO (CRD420261338178). Due to methodological heterogeneity, the findings were synthesised narratively in accordance with SWiM guidance. **Results**: Ten studies, including 1320 adults with OCD, were included. These studies were conducted across five countries between 2010 and 2025. Negative beliefs about thought uncontrollability and danger (MCQ-30 NB) showed the broadest associations across the synthesis, particularly with the checking/harm avoidance and the unacceptable thought dimensions. Thought-fusion constructs (TAF/TFI) were most consistently associated with checking/harm avoidance and unacceptable thought presentations. Beliefs about rituals and stop-signal criteria (BARI/SSQ) were most relevant to symmetry/ordering. In contrast, contamination/washing and hoarding exhibited weaker and less consistent metacognitive profiles. **Conclusions**: The available evidence suggests that metacognitive belief profiles in OCD are not dimensionally uniform. Harm-relevant metacognitions appear to be the most salient factor in the checking and unacceptable thought dimensions, whereas ritual-regulation metacognitions appear to be more relevant to the symmetry/ordering dimension. While these findings may inform dimension-sensitive case formulation and generate testable hypotheses for future metacognitive and exposure-based treatment research, they do not yet justify prescriptive, dimension-specific treatment algorithms. The evidence base remains predominantly cross-sectional and methodologically diverse.

## 1. Introduction

Obsessive–compulsive disorder (OCD) is a disabling and clinically heterogeneous condition. Despite the proven effectiveness of exposure and response prevention (ERP) therapy and selective serotonin reuptake inhibitors (SSRIs), many patients do not achieve complete and sustained remission of symptoms [1,2]. This treatment gap has stimulated interest in identifying the cognitive and metacognitive mechanisms that underpin OCD and its various manifestations.

A growing body of evidence supports the importance of distinguishing OCD symptom dimensions rather than treating the disorder as a unitary construct. Factor-analytic research has identified at least five symptom dimensions with distinct phenomenological features, neurobiological correlates and partially different responses to treatment: (1) contamination/washing, (2) checking/harm avoidance, (3) intrusive thoughts about unacceptable (taboo) topics, (4) symmetry/ordering/repeating, and (5) hoarding [2,3,4]. Treatment response rates differ across these dimensions, with the symmetry/ordering and unacceptable thoughts subtypes showing a somewhat lower response to standard ERP than the contamination or checking subtypes [5,6]. Therefore, understanding which cognitive mechanisms are dimension-specific versus transdiagnostic is directly relevant to personalising treatment.

Metacognitive theory, as formalised by Wells in the Self-Regulatory Executive Function (S-REF) model [7,8], posits that psychological disorders are maintained not by the content of intrusive thoughts per se, but by the meta-level beliefs and strategies individuals apply to them. In OCD, Wells and his colleagues have identified three key metacognitive domains: (a) ‘thought-fusion’ beliefs (the conviction that thoughts have real-world properties or consequences; this is operationalised as thought-action fusion, thought-event fusion or thought-object fusion); (b) beliefs about rituals (positive and negative appraisals of the effects of compulsive behaviour; these are assessed using the Beliefs About Rituals Inventory); and (c) beliefs about stop signals (the metacognitive rules governing when a compulsive sequence may be terminated; these are assessed using the Stop Signals Questionnaire) [9,10]. These three OCD-specific domains are complemented by the more general metacognitive domains measured by the Metacognitions Questionnaire-30 (MCQ-30), which includes negative beliefs about thought uncontrollability and danger (NB), positive beliefs about worry (PB), cognitive confidence (CC), need for cognitive control (NFC) and cognitive self-consciousness (CSC) [11].

Although the metacognitive model of OCD has accumulated substantial empirical support, with metacognitive beliefs demonstrating incremental predictive validity over and above general cognitive beliefs (e.g., inflated responsibility, overestimation of threat and perfectionism) [12,13], one critical question remains unanswered: do specific metacognitive domains exhibit different associations with particular OCD symptom dimensions in adult clinical populations? If such differential profiles exist, they would have direct implications for the delivery of treatment. For example, thought-fusion challenges could be prioritised in cases involving checking or harm avoidance, while stop-signal beliefs could be targeted in cases involving symmetry or ordering.

Conversely, if metacognitive associations are uniform across dimensions, this would support the transdiagnostic application of metacognitive therapy without dimension-specific adaptation.

Previous narrative reviews have examined the metacognitive model of OCD [14,15], but have not systematically mapped metacognitive domains to specific symptom dimensions in adult clinical samples. No published systematic review has addressed this question. The present study therefore conducted a systematic review, preregistered on PROSPERO, aiming to (1) synthesise evidence on the associations between validated metacognitive instruments and OCD symptom dimensions in adult clinical populations and (2) explore the potential clinical implications of any dimension-specific metacognitive profiles for case formulation and treatment planning.

## 2. Materials and Methods

### 2.1. Protocol and Registration

This systematic review was conducted and reported in accordance with the Preferred Reporting Items for Systematic Reviews and Meta-Analyses (Supplementary Material PRISMA 2020 Checklist) statement [16]. Prior to commencement of data extraction, the review was preregistered on PROSPERO (Centre for Reviews and Dissemination, University of York) with registration number CRD420261338178.

### 2.2. Eligibility Criteria

Eligibility was defined according to the PICOS framework:

- Population: Adults (aged ≥ 18) with a primary diagnosis of OCD, as confirmed by a structured clinical interview (SCID or MINI), or by explicit confirmation of the diagnostic criteria in the DSM-IV, DSM-5 or ICD-10/11. Studies must include a minimum of 10 participants with OCD. Non-clinical analogues and student samples without a confirmed OCD diagnosis were excluded.
- Intervention/exposure: Administration of at least one validated metacognitive instrument, including MCQ-30, MCQ-65, TFI, TAF Scale, BARI, SSQ or OBQ-based subscales relevant to the pre-specified belief domains. Studies using only general cognitive (non-metacognitive) measures without a relevant metacognitive component were excluded. To improve conceptual clarity, we made a distinction between general metacognitive measures (MCQ-30/65), measures specific to OCD (TAF/TFI, BARI, SSQ) and belief measures based on the OBQ. Only OBQ-derived constructs were retained as conceptually adjacent belief domains when a primary study used them to operationalise appraisals relating to the meaning, responsibility or control of intrusive thoughts. However, their findings were interpreted more cautiously than those from the core metacognitive measures.
- Comparator: A comparison of metacognitive beliefs across OCD symptom dimensions, as measured by validated dimensional instruments such as: OCI-R subscales, Y-BOCS symptom checklist (dimensional breakdown), DOCS, MOCI subscales, Padua Inventory subscales or D-YBOCS. Studies reporting only a total OCD severity score without a dimensional breakdown were excluded.
- Outcomes: The primary outcome was the reported associations (e.g., correlations, regression coefficients or other effect estimates) linking specific metacognitive belief domains to specific OCD symptom dimensions. Secondary outcome: Evidence on whether metacognitive profiles were associated with dimension-relevant treatment response in studies that included a treatment component.
- Study design: All quantitative designs were eligible, including cross-sectional, case–control, longitudinal, prospective cohort, randomised controlled trials (RCTs), non-randomised controlled trials, open trials and case series (*n* ≥ 10). Qualitative studies, case reports (*n* < 10), editorials and conference abstracts without accessible full data were excluded.

### 2.3. Search Strategy

Four databases were searched without date or language restrictions: PubMed/MEDLINE, Scopus, Cochrane CENTRAL and Google Scholar. The searches were conducted in March 2026. The following three-block Boolean search strategy was applied:

PubMed/MEDLINE search string:

Block 1 (OCD): (“obsessive-compulsive disorder”[MeSH Terms] OR “obsessive-compulsive disorder”[Title/Abstract] OR “OCD”[Title/Abstract] OR “obsessive compulsive”[Title/Abstract]).

Block 2 (Metacognition): AND (“metacognit*”[Title/Abstract] OR “metacognitions questionnaire”[Title/Abstract] OR “MCQ-30”[Title/Abstract] OR “MCQ-65”[Title/Abstract] OR “thought fusion”[Title/Abstract] OR “thought-action fusion”[Title/Abstract] OR “TAF”[Title/Abstract] OR “beliefs about rituals”[Title/Abstract] OR “BARI”[Title/Abstract] OR “stop signal”[Title/Abstract] OR “obsessive beliefs questionnaire”[Title/Abstract] OR “OBQ”[Title/Abstract] OR “metacognitive beliefs”[Title/Abstract] OR “metacognitive therapy”[Title/Abstract]).

Block 3 (Dimensions): AND (“symptom dimension*”[Title/Abstract] OR “symptom subtype*”[Title/Abstract] OR “OCD subtype*”[Title/Abstract] OR “contamination”[Title/Abstract] OR “washing”[Title/Abstract] OR “checking”[Title/Abstract] OR “hoarding”[Title/Abstract] OR “symmetry”[Title/Abstract] OR “ordering”[Title/Abstract] OR “intrusive thought*”[Title/Abstract] OR “taboo thought*”[Title/Abstract] OR “OCI-R”[Title/Abstract] OR “Y-BOCS”[Title/Abstract] OR “DOCS”[Title/Abstract]).

Equivalent Boolean logic was used for Scopus and Cochrane CENTRAL searches, adapted to each platform’s syntax. Google Scholar was searched in three targeted passes, with the first 200 results sorted by relevance being examined in each pass. Additional strategies included tracking the backward and forward citations of all included articles, as well as targeted author searches (Wells, Fisher, Solem, Myers, Abramowitz and Simons) in PubMed.

### 2.4. Study Selection and Data Extraction

Records were imported into Zotero and automatically deduplicated, with manual verification. Two reviewers screened all titles and abstracts independently against the PICOS eligibility criteria, resolving any disagreements by consensus or third-reviewer adjudication. Full texts were retrieved for all potentially eligible records and reassessed against the full eligibility criteria. The reasons for excluding records at the full-text stage were recorded. A standardised data extraction form was used to capture the following information: study identification; design; sample characteristics (N, age, sex, diagnosis method); OCD severity instrument and score; OCD dimensional instrument(s) and subscale scores; metacognitive instrument(s) and subscale scores; all reported statistical associations (r, β, OR, *p*-value); covariates controlled; and country and setting. The study-level analytical characteristics and the covariates/statistical controls are summarised in Supplementary Table S1.

### 2.5. Quality Assessment Method

The risk of bias was assessed using tools that were matched to the study design. These were: the Newcastle–Ottawa Scale—Cross-Sectional Adaptation (NOS-CS) for cross-sectional studies; the standard Newcastle–Ottawa Scale (NOS) for longitudinal studies; the Cochrane Risk of Bias 2.0 (RoB 2.0) tool for randomised controlled trials (RCTs); and the Joanna Briggs Institute (JBI) Case Series Appraisal tool for open trials. Two reviewers conducted the assessments independently and resolved any disagreements by consensus. Studies were not excluded based on quality alone. Instead, quality ratings were used to provide context for interpretation.

### 2.6. Data Synthesis

Due to the methodological and clinical heterogeneity of the included studies, which encompassed different metacognitive instruments, dimensional measures, sample compositions, and statistical approaches, a pooled meta-analysis was not pursued as the primary synthesis method. Instead, in accordance with the Synthesis Without Meta-Analysis (SWiM) guidelines [17], we conducted a structured narrative synthesis, organising the evidence according to the five symptom clusters specified in the protocol and, within each cluster, by metacognitive domain.

Where primary studies used different dimensional labels or instruments, the findings were harmonised using explicit mapping rules. For example, ‘washing’ and ‘cleaning’ labels were mapped to ‘contamination’ and ‘washing’; ‘checking’, ‘doubting’, and ‘responsibility/harm’ labels were mapped to ‘checking’ and ‘harm avoidance’; ‘obsessing’, ‘taboo/unacceptable thoughts’, and ‘neutralising phenomena’ were mapped to ‘unacceptable thoughts’ when conceptually linked to ‘intrusive taboo thoughts’; ‘ordering/organising/repeating’ labels were mapped to ‘symmetry/ordering’; and ‘hoarding’ was retained as a separate cluster. Original study terminology was retained in Table 1 for transparency.

Convergence of findings was summarised using a descriptive consistency framework rather than a formal evidence-grading system: consistent (≥2 studies in the same direction), mixed (contradictory directions across studies) or insufficient (<2 studies or sparse/heterogeneous evidence). The threshold of ≥2 studies was used only as an indicator of minimal replication across independent samples, and does not integrate effect size, sample precision or risk of bias into a single weighted score. These descriptive ratings were therefore interpreted alongside study design, risk of bias, analytic approach and covariate/statistical adjustment. Two summary tables were produced: Table 1 details the characteristics of the studies and Table 2 presents a descriptive evidence matrix mapping the core metacognitive domains to the symptom dimensions. OBQ-based findings were included in Table 1 and the narrative synthesis as conceptually adjacent domains; however, they were not used to expand the core matrix columns in Table 2. Study-level analytic characteristics and covariate/statistical controls are summarised in Supplementary Table S1.

## 3. Results

### 3.1. Search Results and Study Selection

**Figure 1:**
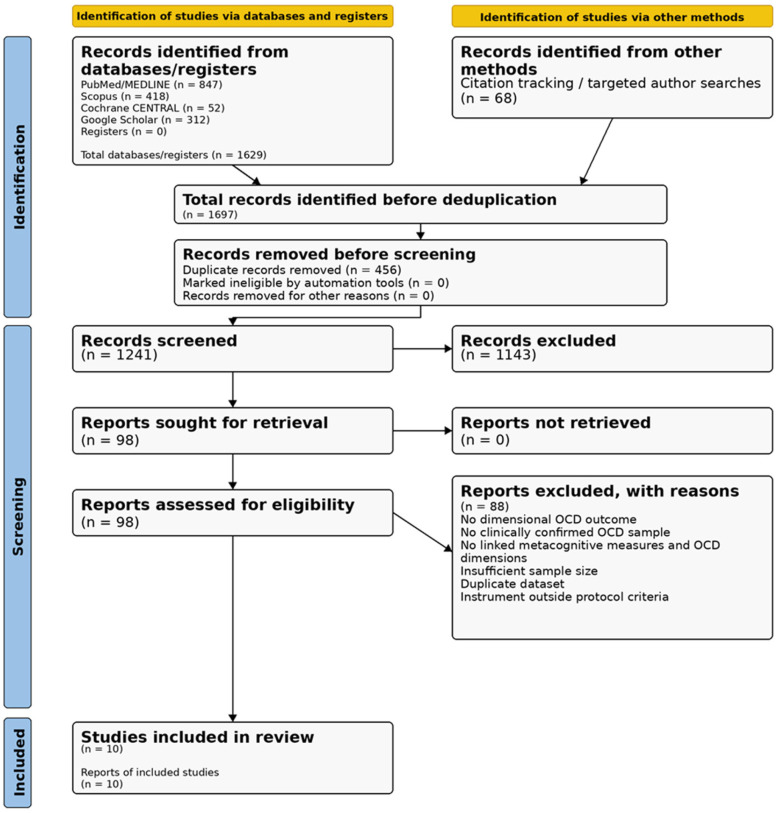
PRISMA 2020 flow diagram showing the process of selecting studies. OCD = obsessive–compulsive disorder. The inter-rater κ value for title/abstract screening was 0.82 (95% CI: 0.76–0.88).

The combined search of PubMed (*n* = 847), Scopus (*n* = 418), Cochrane CENTRAL (*n* = 52), Google Scholar (*n* = 312) and citation tracking (*n* = 68) yielded 1697 records. After removing 456 duplicates, 1241 unique records were screened by title and abstract, with 1143 being excluded at this stage. A total of 98 reports were assessed in full. Of these, 88 were excluded, leaving 10 studies that met the eligibility criteria for inclusion in the narrative synthesis. The main reasons for excluding the full texts were the absence of a dimensional OCD outcome, a lack of a clinically confirmed OCD sample, failure to link metacognitive measures to symptom dimensions in the analysis, an insufficient OCD sample size, duplicate datasets or the use of instruments outside the pre-specified criteria. Inter-rater agreement at the title/abstract screening stage was κ = 0.82 (95% CI: 0.76–0.88). The PRISMA 2020 flow diagram is presented in Figure 1.

### 3.2. Characteristics of Included Studies

The ten included studies enrolled 1320 adults with OCD, with sample sizes for the OCD group ranging from 24 to 562. The studies were conducted in Republic of Korea, Turkey, Norway, Germany and India between 2010 and 2025 (see Table 1). While most studies were cross-sectional, the evidence base also included repeated-measures and treatment-oriented designs. The MCQ-30 was the most frequently used metacognitive measure, and subsets of studies also used the TFI/TAFS, BARI, SSQ and OBQ-based belief measures. OCD dimensions were mainly assessed using OCI-R-derived subscales, with additional use of MOCI, DOCS, D-YBOCS and Y-BOCS/Padua-based dimensional assessments. As diagnostic procedures and analytic models varied across reports, this should be considered when comparing findings across instruments and symptom clusters. A more detailed summary of the study-level analytical approach and the covariate and statistical adjustments applied across the studies can be found in Supplementary Table S1.

### 3.3. Quality Assessment

Quality ratings for the non-randomised studies ranged from six to eight out of ten on the adapted Newcastle–Ottawa framework, whereas the single pilot randomised controlled trial (RCT) was judged to be at low risk of bias on RoB 2.0 (Table 1). Common concerns across the observational literature included limited adjustment for depression or anxiety, convenience sampling, modest sample sizes and the absence of a priori power calculations. Repeated-measures and treatment-focused reports were also constrained by incomplete follow-up reporting and the secondary nature of some dimension-specific analyses. No study was excluded on quality grounds.

### 3.4. Metacognitive Associations by OCD Symptom Dimension

#### 3.4.1. Checking/Harm Avoidance

Throughout the synthesis, the checking/harm avoidance cluster demonstrated the most consistent and significant metacognitive associations. Negative beliefs about the uncontrollability and danger of thought (MCQ-30 NB) were repeatedly linked with the checking/harm avoidance cluster. Thought-fusion measures (TAF/TFI) provided the strongest additional support for this cluster. OCD-specific ritual-regulation measures (BARI and, in some analyses, SSQ) also showed positive associations, albeit less consistently. Overall, this dimension showed the strongest convergence across instruments and studies.

#### 3.4.2. Unacceptable (Taboo) Thoughts

The cluster of unacceptable thoughts also showed a relatively coherent metacognitive profile. Thought-fusion constructs emerged most consistently, particularly in studies that assessed the moral or likelihood components of TAF. NB again showed repeated positive associations. Several studies found that OCD-specific measures focusing on rituals or stop rules tracked features mapped to the cluster of unacceptable thoughts (e.g., obsessing or neutralising subscales in the original studies). This suggests that unacceptable intrusive thoughts may occur alongside metacognitive evaluations of the importance of thoughts and the perceived need to neutralise them.

#### 3.4.3. Symmetry/Ordering

The symmetry/ordering cluster exhibited a somewhat different profile. Compared with the checking/harm avoidance and unacceptable thoughts clusters, fusion measures were less consistent. However, stop-signal beliefs (SSQ) and beliefs about rituals (BARI) appeared to be more relevant. This pattern is conceptually compatible with the phenomenology of ‘just-right’ experiences and difficulty in stopping rituals. Some studies also reported associations with positive beliefs about worry and cognitive self-consciousness. However, these findings were less extensive than those for the SSQ/BARI and should be interpreted cautiously. Overall, the findings for the symmetry/ordering cluster suggest that this dimension may be characterised less by the perceived power of thoughts themselves, and more by the metacognitive rules that govern the continuation, monitoring and completion of ritual sequences. Nevertheless, the number of contributing studies remained modest.

#### 3.4.4. Contamination/Washing

Contamination/washing exhibited a weaker and less consistent pattern. Although broad correlations with general metacognitive measures, particularly NB, were sometimes reported at the bivariate level, these associations were not consistently retained in more stringent models. Across the available studies, fusion-based measures often appeared to be less informative for contamination/washing than for checking/harm avoidance or unacceptable thoughts, and were often non-significant. Consequently, the contamination/washing cluster appeared to be the least well captured by the current set of thought-focused metacognitive instruments. Alternatively, it may depend more strongly on mechanisms that are not directly measured by most of the included studies. Therefore, the available evidence only supports cautious inferences for this dimension.

#### 3.4.5. Hoarding

Of all the symptom clusters examined, hoarding showed the most heterogeneous results and was represented in relatively few analyses. Where examined, metacognitive variables tended to display weaker or less specific associations than those observed for checking/harm avoidance, unacceptable thoughts or symmetry/ordering. Therefore, the evidence for hoarding should be regarded as preliminary due to the small number of informative studies, and because hoarding-related findings were often secondary analyses rather than the primary focus of the included reports.

### 3.5. Evidence Matrix

Table 2 summarises the evidence matrix, which presents the consistency and direction of the associations between the metacognitive domains and the OCD symptom dimensions across the included studies.

## 4. Discussion

This systematic review suggests that the association between metacognitive beliefs and OCD symptoms varies across different dimensions. The most consistent findings across different countries, measurement tools and general and OCD-specific metacognitive measures were the associations between checking/harm avoidance and negative beliefs about uncontrollability/danger (NB), and between thought fusion and unacceptable thoughts. By contrast, symmetry/ordering exhibited a more specific ritual-regulation profile that appeared to depend more strongly on OCD-specific measures, such as the BARI and SSQ. Contamination/washing and hoarding, however, were less consistently explained and more susceptible to measurement and analytical heterogeneity.

### 4.1. Harm-Relevant Metacognitions and the Checking/Harm Avoidance Dimension

The most consistent pattern was related to checking/harm avoidance, for which NB and thought-fusion measures were consistently identified as relevant across studies using different dimensional instruments. This is consistent with the S-REF account, in which intrusions become clinically significant when appraised as dangerous, uncontrollable or likely to cause harm [8,9]. However, as most of the data were cross-sectional, these findings should be interpreted as robust associations rather than as proof of a causal sequence.

The additional contribution of TAF/TFI to this dimension is also consistent with the clinical phenomenology of harm-related obsessions and checking rituals, in which perceived responsibility and the feared consequences of thinking about harm are central themes. However, the size and independence of this contribution varied across studies and instruments. Therefore, the present review supports convergence at the level of pattern rather than a single quantitative estimate.

### 4.2. Fusion Beliefs and the Unacceptable Thoughts Dimension

The dimension of unacceptable thoughts showed a second comparatively coherent profile. Beliefs based on fusion, especially those concerning moral equivalence or the significance of unwanted thoughts, appeared particularly relevant. This is clinically plausible, as taboo intrusions can be distressing not only because of their content, but also because of the meaning attributed to having them. Nevertheless, as the studies differed in their measures and statistical models, any hierarchy among specific fusion constructs should be considered provisional.

### 4.3. Ritual-Regulation Metacognitions and the Symmetry/Ordering Dimension

The symmetry/ordering findings were notable because they indicated a weaker link with fusion constructs and a stronger link with stop-signal and ritual-regulation beliefs. This distinction aligns with the clinical observation that many symmetry/ordering presentations are characterised by a sense of incompleteness, experiences of things being ‘not quite right’, and difficulty in determining when an action has been satisfactorily completed [28,29]. The current synthesis therefore suggests that this cluster involves an emphasis on metacognitive monitoring and termination rules rather than thought-action equivalence alone. Some studies have also linked symmetry/ordering to positive beliefs about worry or increased cognitive self-consciousness. While these findings are theoretically interesting, they were not replicated as consistently as the SSQ/BARI pattern and should therefore be treated as tentative.

### 4.4. The Contamination/Washing and Hoarding Dimensions: Metacognitive Underrepresentation

The relatively weak signals for contamination/washing and hoarding can be interpreted in at least two non-mutually exclusive ways. First, these dimensions may rely less on the metacognitive constructs captured by the MCQ, TAFS/TFI, BARI and SSQ. Second, the available instruments may not adequately capture the mechanisms most relevant to these presentations. Contamination/washing, disgust sensitivity and threat appraisal may play a larger role, while hoarding, attachment, indecisiveness and information-processing factors may be at least as important as thought-focused metacognitions [23,27,30,31,32,33]. Accordingly, the current review does not support the idea that metacognitive processes are unimportant in these dimensions. Rather, it suggests that the current evidence base is comparatively sparse and that the metacognitive measures currently in use are not particularly informative.

### 4.5. Clinical Implications

**Figure 2:**
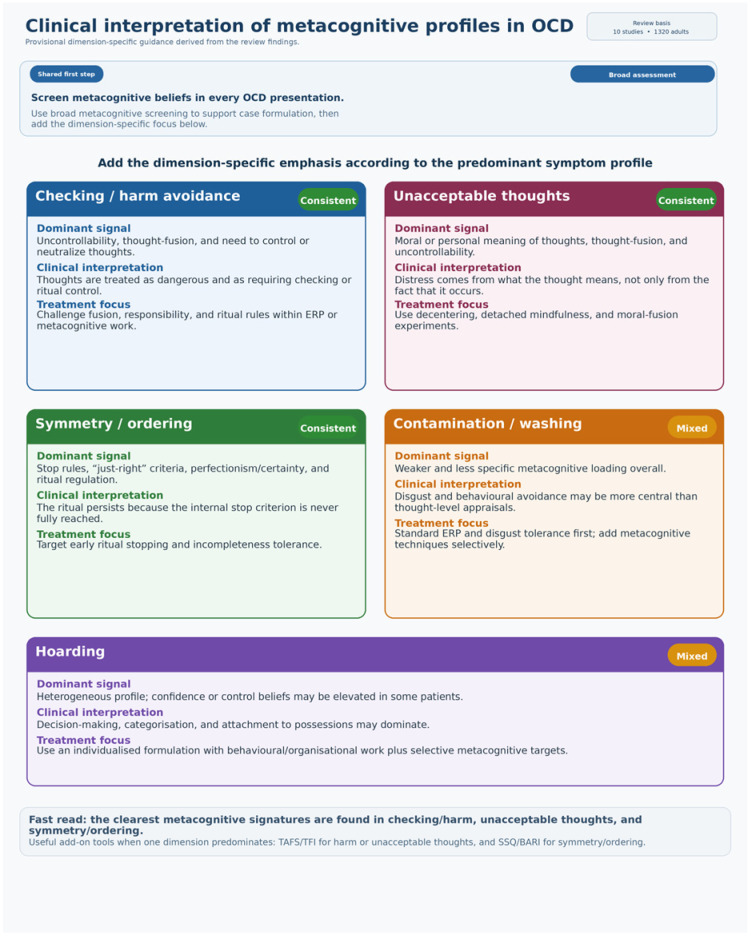
Provisional clinical translation of dimension-specific metacognitive profiles in OCD, derived from narrative synthesis. This figure summarises where recurrent metacognitive signals were strongest, and where evidence was mixed or sparse. It should be interpreted as an aid to case formulation rather than as a validated treatment algorithm.

These findings should be regarded as provisional and hypothesis-generating with regard to their clinical implications. The current evidence base is insufficient to support prescriptive, dimension-specific treatment algorithms. At most, the synthesis suggests that, when formulating cases individually, it may be useful to consider whether beliefs about uncontrollability/danger and thought fusion are particularly relevant in cases involving checking/harm avoidance and unacceptable thoughts, and whether stop-signal or ritual-completion beliefs are more pertinent in cases involving symmetry/ordering. Further prospective and comparative treatment research is required before these possibilities can be translated into specific modifications of metacognitive therapy (MCT) or exposure and response prevention (ERP). For contamination/washing and hoarding, broader formulations may be required as current instruments may not adequately capture the most relevant mechanisms for these dimensions. Figure 2 should therefore be interpreted as a heuristic summary and case-formulation aid only, not as a validated treatment algorithm.

### 4.6. Limitations

Several limitations should be noted. First, the evidence base was predominantly cross-sectional; only one pilot randomised trial, one repeated-measures treatment study and one report with short prospective follow-up were identified. Therefore, temporal precedence and treatment responsiveness cannot be inferred with confidence. Second, substantial heterogeneity was present at multiple levels, including dimensional measures, metacognitive instruments, covariate adjustment and statistical models. This constrained direct comparability and precluded meta-analysis. Third, the synthesis involved harmonising non-identical symptom scales into a common five-cluster framework. While this enhanced the interpretability of the data, some studies examined constructs that overlapped to a certain extent (e.g., obsessing versus having unacceptable thoughts; organising versus having a need for symmetry/order), which introduced an element of judgement into the classification process. Fourth, measurement comparability was limited by the use of general metacognitive scales, OCD-specific instruments and OBQ-based measures. One of the included studies used the legacy OBQ-87 rather than the brief OBQ-44 format. Fifth, database coverage did not include PsycINFO or Embase, and Google Scholar screening relied on targeted searches rather than exhaustive platform coverage. Sixth, several studies had modest sample sizes and dimension-specific analyses were sometimes secondary or exploratory rather than incorporating clearly pre-specified primary endpoints. In addition, the evidence matrix should not be interpreted as an assessment of certainty. Publication bias and selective outcome reporting cannot be ruled out. However, a formal assessment was not feasible due to the small number of studies, the variety of designs and the lack of comparable effect estimates across syntheses. The geographic representation was also limited, with only studies from Republic of Korea, Turkey, Norway, Germany and India identified, and none from the Western Hemisphere or low-income settings.

### 4.7. Future Research

Future studies should employ preregistered and explicitly defined mapping rules, standardised dimensional measures and adequately powered longitudinal or experimentally informative designs. Specific hypotheses worth testing include:

- Whether baseline negative beliefs about uncontrollability/danger and thought-fusion constructs prospectively predict persistence, relapse, or treatment resistance in checking/harm avoidance and unacceptable thought presentations;
- Whether beliefs indexed by BARI and SSQ predict difficulty terminating rituals and differential treatment response in symmetry/ordering presentations;
- Whether change in dimension-relevant metacognitive beliefs mediates response to metacognitive therapy or exposure and response prevention. Future instrument development may also need to expand the scope of assessment beyond current thought-focused constructs in order to capture disgust-related appraisals in contamination/washing and attachment-, indecisiveness-, and decision-related processes in hoarding. Cross-cultural validation in underrepresented regions, including the Western Hemisphere and lower-resource settings, remains essential.

## 5. Conclusions

This systematic review suggests that metacognitive belief profiles in OCD are not uniform across the board. The clearest recurring associations were with negative beliefs about uncontrollability/danger and thought-fusion constructs in presentations of checking/harm avoidance and of unacceptable thoughts, whereas ritual-regulation beliefs were comparatively more relevant to presentations of symmetry/ordering. In contrast, contamination/washing and hoarding were supported by weaker and more heterogeneous evidence. While these findings may inform dimension-sensitive case formulation and suggest priorities for future personalised treatment research, they should not yet be used to create prescriptive, dimension-specific treatment algorithms. Further rigorous longitudinal and comparative intervention studies are required before dimension-specific metacognitive targeting can be confidently recommended.

## Figures and Tables

**Table 1:** Characteristics of included studies, ordered chronologically from most recent to oldest.

Study	Country	Sample	Design	OCD Measure(s)	Metacognitive Measure(s)	Symptom Dimensions Assessed	Quality/RoB
Güneysu et al. (2025) [18]	Turkey	106 (+200 HC)	Cross-sectional	OCI-R	TFI, BARI, SSQ, OBQ-44	Washing, checking, organising, obsessing, hoarding, neutralising	7/10
Glombiewski et al. (2021) [19]	Germany	24	Pilot randomised controlled trial (MCT vs. ERP)	Y-BOCS, PI-R	TAF, BARI, SSQ, MCQ-30	Dimensional change scores; total OCD severity	Low risk (RoB 2.0)
Kim et al. (2021) [20]	Republic of Korea	562 (+236 HC)	Cross-sectional + 3-month pharmacological follow-up	OCI-R-K, Y-BOCS	MCQ-30	Washing, checking, obsessing, neutralising, ordering, hoarding	8/10
Kim and Lee (2020) [21]	Republic of Korea	65 (+45 HC)	Cross-sectional	DOCS, OCI-R	TAFS, OBQ-44 (metacognitive subscale)	Contamination, responsibility/harm, unacceptable thoughts, symmetry	7/10
Tümkaya et al. (2018) [22]	Turkey	51	Cross-sectional	MOCI	MCQ-30	Checking, cleaning, slowness, doubting, rumination	7/10
Myers et al. (2017) [23]	Norway	210	Cross-sectional	OCI-R, Y-BOCS-SR	TFI, BARI, SSQ, MCQ-30	Washing, checking, obsessing, neutralising, ordering, hoarding	8/10
Cordeiro et al. (2015) [24]	India	75	Cross-sectional	D-YBOCS	OBQ-87 (metacognitive subscales)	Contamination, symmetry, sexual/religious, aggression, somatic, hoarding	6/10
Grøtte et al. (2015) [25]	Norway	83	Repeated-measures treatment study	OCI-R, Y-BOCS	MCQ-30, OBQ-44	All OCI-R subscales; pre–post change scores	7/10
Timpano et al. (2014) [26]	Germany	73 *	Cross-sectional	OCI-R	MCQ-30	Obsessing, ordering, checking, washing, hoarding	6/10
Solem et al. (2010) [27]	Norway	71 **	Cross-sectional	OCI-R, Y-BOCS	TFI, BARI, SSQ, MCQ-30	Washing, checking, obsessing, neutralising, ordering	7/10

* OCD-diagnosed subgroup of a larger young-adult sample (*n* = 160). ** Clinical OCD arm of a larger patient cohort. Sample: OCD sample is listed first; comparator groups are shown in parentheses when present. Quality/RoB: x/10 = adapted Newcastle–Ottawa score for observational studies (higher scores indicate better methodological quality); “Low risk (RoB 2.0)” = low risk of bias on the Cochrane RoB 2.0 tool for the pilot randomised trial. Abbreviations: BARI = Beliefs About Rituals Inventory; D-YBOCS = Dimensional Yale–Brown Obsessive–Compulsive Scale; DOCS = Dimensional Obsessive–Compulsive Scale; ERP = exposure and response prevention; HC = healthy controls; MCT = metacognitive therapy; MCQ-30 = Metacognitions Questionnaire-30; MOCI = Maudsley Obsessive–Compulsive Inventory; OBQ = Obsessive Beliefs Questionnaire; OCI-R = Obsessive–Compulsive Inventory-Revised; OCI-R-K = Korean version of the OCI-R; PI-R = Padua Inventory-Revised; SSQ = Stop Signals Questionnaire; TAF(S) = Thought-Action Fusion (Scale); TFI = Thought-Fusion Instrument; Y-BOCS = Yale–Brown Obsessive–Compulsive Scale; Y-BOCS-SR = Self-Report Yale–Brown Obsessive–Compulsive Scale. The original study labels are preserved in this table. In the narrative synthesis, ‘obsessing/taboo thoughts/neutralising’ were harmonised under ‘unacceptable thoughts’ where they were conceptually linked to ‘intrusive taboo thoughts’, and ‘organising/repeating’ labels were harmonised under ‘symmetry/ordering’. OBQ-based measures are shown as reported by the primary studies, but should be interpreted more cautiously as conceptually adjacent belief domains rather than core metacognitive measures.

**Table 2:** Descriptive evidence matrix of associations between core metacognitive domains and OCD symptom dimensions.

OCD Symptom Dimension	MCQ-30 Domains					OCD-Specific Metacognitive Measures		
	NB	PB	CC	NC	CSC	TAF/TFI	BARI	SSQ
Checking/harm avoidance	C+, *n* = 4	M, *n* = 3	—	—	—	C+, *n* = 4	C+, *n* = 3	C+, *n* = 2
Unacceptable thoughts	C+, *n* = 4	M, *n* = 2	—	—	—	C+, *n* = 4	C+, *n* = 3	M, *n* = 2
Symmetry/ordering	M, *n* = 3	C+, *n* = 2	C+, *n* = 2	M, *n* = 2	C+, *n* = 2	C0, *n* = 2	C+, *n* = 2	C+, *n* = 3
Contamination/washing	M, *n* = 4	M, *n* = 2	—	—	—	C0, *n* = 3	M, *n* = 2	M, *n* = 2
Hoarding	I, *n* = 3	I, *n* = 2	M, *n* = 2	M, *n* = 2	—	I, *n* = 2	I, *n* = 2	I, *n* = 2

Cells report the descriptive judgement, followed by the number of informative studies (*n*). C+ indicates a consistent positive association, C0 indicates a consistent null or no clear association, M indicates mixed findings, and I indicates insufficient evidence. This matrix is intended to provide a descriptive overview of the narrative synthesis rather than forming the basis of a weighted certainty framework. Effect size, sample precision and risk of bias are not combined into a single summary score and should be considered alongside Table 1, the main text and Supplementary Table S1. OBQ-based findings are discussed narratively, rather than being shown in separate columns, because they were considered conceptually related to, rather than part of, the core metacognitive domains.

## Data Availability

No new datasets were created or analysed in this study. All data were extracted from the cited published studies and are reported within this manuscript.

## Supplementary Materials

The following supporting information can be downloaded at: https://www.mdpi.com/article/10.3390/jcm15103586/s1, PRISMA 2020 Checklist; Supplementary Table S1: Analytic approach and covariate/statistical adjustment across included studies.

## Abbreviations

The following abbreviations are used in this manuscript:

BARI	Beliefs About Rituals Inventory
CC	cognitive confidence
CENTRAL	Cochrane Central Register of Controlled Trials
CI	confidence interval
CSC	cognitive self-consciousness
D-YBOCS	Dimensional Yale–Brown Obsessive–Compulsive Scale
DOCS	Dimensional Obsessive–Compulsive Scale
DSM-5	Diagnostic and Statistical Manual of Mental Disorders, Fifth Edition
DSM-IV	Diagnostic and Statistical Manual of Mental Disorders, Fourth Edition
ERP	exposure and response prevention
HC	healthy controls
ICD-10/11	International Classification of Diseases, 10th/11th revision
JBI	Joanna Briggs Institute
MCQ-30	Metacognitions Questionnaire-30
MCQ-65	Metacognitions Questionnaire-65
MCT	metacognitive therapy
MEDLINE	Medical Literature Analysis and Retrieval System Online
MINI	Mini-International Neuropsychiatric Interview
MOCI	Maudsley Obsessive–Compulsive Inventory
NB	negative beliefs about uncontrollability and danger
NC	need to control thoughts
NOS	Newcastle–Ottawa Scale
NOS-CS	Newcastle–Ottawa Scale—Cross-Sectional Adaptation
OBQ	Obsessive Beliefs Questionnaire
OBQ-44	44-item Obsessive Beliefs Questionnaire
OBQ-87	87-item Obsessive Beliefs Questionnaire
OCD	obsessive–compulsive disorder
OCI-R	Obsessive–Compulsive Inventory-Revised
OCI-R-K	Korean version of the Obsessive–Compulsive Inventory-Revised
OR	odds ratio
PB	positive beliefs about worry
PI-R	Padua Inventory-Revised
PICOS	Population, Intervention/Exposure, Comparator, Outcomes, and Study Design
PRISMA	Preferred Reporting Items for Systematic Reviews and Meta-Analyses
PROSPERO	International Prospective Register of Systematic Reviews
RCT	randomised controlled trial
RoB	risk of bias
RoB 2.0	Cochrane Risk of Bias 2.0 tool
S-REF	Self-Regulatory Executive Function
SCID	Structured Clinical Interview for DSM Disorders
SSQ	Stop Signals Questionnaire
SSRIs	selective serotonin reuptake inhibitors
SWiM	Synthesis Without Meta-Analysis
TAF	thought-action fusion
TAFS	Thought-Action Fusion Scale
TFI	Thought-Fusion Instrument
Y-BOCS	Yale–Brown Obsessive–Compulsive Scale
Y-BOCS-SR	Self-Report Yale–Brown Obsessive–Compulsive Scale
