# Supplementary material for: Metacognitive Belief Profiles Across OCD Symptom Dimensions: A Systematic Review and Clinical Implications for Personalised Treatment
Source: J Clin Med. 2026 May 7;15(10):3586. doi: 10.3390/jcm15103586 (PMC13208003; doi:10.3390/jcm15103586)
Supplement: Supplementary file 1 [file jcm-15-03586-s001.zip › jcm-4279527-Supplementary Table S1.pdf]

Supplementary Table S1. Analytic approach and covariate/statistical adjustment across included studies

Study	OCD dimensional measure(s) used in the synthesis	Metacognitive measure(s)	Main analytic approach reported	Covariates/statistical controls reported	Depression/anxiety adjusted?	Notes for interpretation
Güneysu et al. (2025)	OCI-R	TFI; BARI; SSQ; OBQ-44	Group comparisons (χ^2, t test/Mann–Whitney U), Pearson correlations, linear regression, and hierarchical linear regression.	Hierarchical model: Step 1 PSWQ (worry); Step 2 OBQ subscales (PIU, responsibility, overestimation of threat); Step 3 TFI, BARI, and SSQ.	No	Tests incremental contribution of OCD-specific metacognitive beliefs beyond worry and OBQ beliefs; no depression/anxiety covariates reported.
Glombiewski et al. (2021)	Y-BOCS; PI-R	TAF; BARI; SSQ; MCQ-30	Pilot randomized comparison of MCT versus ERP, with efficacy analyses at post-treatment and 3-month follow-up; treatment fidelity and credibility also assessed.	Randomized treatment arm/time structure inherent to the trial; study-level covariate adjustment was not reported in the retrieved abstract.	Not reported	Treatment/change study rather than baseline cross-sectional prediction study.
Kim et al. (2021)	OCI-R-K; Y-BOCS	MCQ-30	Baseline MANOVA comparing OCD and controls; Pearson correlations; repeated-measures ANOVA over 3-month pharmacological follow-up.	Age, sex, and MADRS controlled in the MANOVA and repeated-measures ANOVA; expectation–maximization imputation used for <5% missing values.	Depression only (MADRS); no anxiety covariate reported	Mixed cross-sectional and short-term follow-up design; dimensional associations derive mainly from baseline correlations.
Kim and Lee (2020)	DOCS; OCI-R	TAFS; OBQ-44 responsibility/threat	Zero-order correlations and hierarchical multiple regressions predicting DOCS and OCI symptom dimensions; multicollinearity checked.	Step 1: OBQ-44 responsibility/threat and trait guilt; Step 2: TAFS subscales.	No explicit depression/anxiety adjustment in the main regressions	BDI was measured, but the retrieved regression description does not clearly indicate that it was entered as a covariate. Trait guilt was entered as a non-metacognitive comparator/predictor and is therefore not listed under the metacognitive measures.
Tümkaya et al. (2018)	MOCI	MCQ-30	ANCOVA for OCD versus control comparisons and hierarchical multiple regressions within the OCD group.	ANCOVA controlled HAM-D and HAM-A; hierarchical regression Step 1 age, sex, HAM-D, HAM-A; Step 2 MCQ-30 subscales.	Yes—depression (HAM-D) and anxiety (HAM-A)	MOCI dimensions (checking, cleaning, slowness, doubting, rumination) required harmonization for the five-cluster synthesis.
Myers et al. (2017)	OCI-R; Y-BOCS-SR	TFI; BARI; SSQ	Correlations and hierarchical regressions testing metacognitive domains in their hypothesized order of activation.	Worry and non-metacognitive OCD beliefs linked to other theories were controlled before entry of the metacognitive domains.	No explicit depression/anxiety covariate reported	Clinical OCD sample.
Cordeiro et al. (2015)	D-YBOCS	OBQ-87 belief domains	Backward linear regressions predicting each D-YBOCS symptom dimension.	OBQ belief domains entered as predictors; comorbid anxiety disorders and depressive disorders entered to eliminate confounding; conservative significance threshold ($p \leq 0.01$).	Yes—via comorbid anxiety/depressive diagnoses	Uses OBQ-based belief domains rather than core MCQ or OCD-specific metacognitive instruments.

Study	OCD dimensional measure(s) used in the synthesis	Metacognitive measure(s)	Main analytic approach reported	Covariates/statistical controls reported	Depression/anxiety adjusted?	Notes for interpretation
Grøtte et al. (2015)	OCI-R; Y-BOCS	MCQ-30; OBQ-44 perfectionism/responsibility	Pre/post correlations and logistic regressions predicting clinically significant change/recovery on Y-BOCS and OCI-R.	Baseline symptom severity, baseline BDI, and raw or reliable change scores in perfectionism, responsibility, MCQ-30, and OBQ-44.	Depression only (BDI); no anxiety covariate reported	Treatment/change study; tests whether change in beliefs predicts recovery rather than whether baseline beliefs predict baseline symptom dimensions.
Timpano et al. (2014)	OCI-R symptom dimensions; hoarding symptoms	MCQ-30	Series of linear regressions examining specificity across obsessive-compulsive symptom dimensions and hoarding; additional analyses addressed hoarding-specific beliefs and hoarding symptoms.	General distress was controlled; shared variance across symptom dimensions and hoarding was factored out.	General distress adjusted, but no depression/anxiety-specific covariate identifiable from the retrieved abstract	Only the diagnostically eligible OCD subgroup reported in the primary paper was extracted for the present review; the broader university sample was not used for the narrative synthesis.
Solem et al. (2010)	OCI-R; Y-BOCS-SR	TFI; BARI; SSQ	Study I: correlations and OCD versus control comparisons. Study II: hierarchical regressions testing the metacognitive sequence fusion beliefs → beliefs about rituals → stop signals.	Worry, threat, responsibility, and perfectionism/certainty were controlled before the metacognitive steps in the stricter predictive models.	No explicit depression/anxiety covariate reported	Predictive testing of stop signals was conducted in the community replication sample rather than solely in the clinical OCD sample.

Note. This table is intended to increase transparency about analytic approach and covariate/statistical adjustment; it does not replace the main study-characteristics table.

Abbreviations. ANCOVA, analysis of covariance; BARI, Beliefs About Rituals Inventory; BDI, Beck Depression Inventory; DOCS, Dimensional Obsessive-Compulsive Scale; D-YBOCS, Dimensional Yale-Brown Obsessive-Compulsive Scale; ERP, exposure and response prevention; HAM-A, Hamilton Anxiety Rating Scale; HAM-D, Hamilton Depression Rating Scale; MADRS, Montgomery–Åsberg Depression Rating Scale; MANOVA, multivariate analysis of variance; MCT, metacognitive therapy; MOCI, Maudsley Obsessive-Compulsive Inventory; OBQ, Obsessive Beliefs Questionnaire; OCI-R, Obsessive-Compulsive Inventory-Revised; PI-R, Padua Inventory-Revised; PSWQ, Penn State Worry Questionnaire; SSQ, Stop Signals Questionnaire; TAF(S), Thought-Action Fusion (Scale); TFI, Thought Fusion Instrument; Y-BOCS-SR, Yale-Brown Obsessive-Compulsive Scale Self-Report.
